# The Anti-Inflammatory Effect of Fructus Kochiae on Allergic Contact Dermatitis Rats via pERK1/2/TLR4/NF-*κ*B Pathway Activation

**DOI:** 10.1155/2018/1096920

**Published:** 2018-01-04

**Authors:** Zuoqi Xiao, Suxi Xiao, Yongning Zhang, Tao Pan, Bo Ouyang

**Affiliations:** ^1^Hunan Provincial Maternal and Child Health Care Hospital, Changsha 410008, China; ^2^College of Biomedical Engineering, South-Central University for Nationalities, Wuhan 430074, China

## Abstract

Allergic contact dermatitis (ACD) is a common irritability skin disease, which can be cured by using the Chinese patent medicine. To explore the pharmacological effect of total flavonoids of Fructus Kochiae (FK) on ACD, we used dinitrochlorobenzene- (DNCB-) induced ACD rats. Five groups were used in our experiments. The normal group and the DNCB group were treated with 0.5% CMC-Na; the DNCB + hFK group was treated with a high dose of total flavonoids of FK (200 mg/kg); the DNCB + lFK group was treated with a low dose of FK (100 mg/kg); the DNCB + Pre group was treated with prednisolone acetate (2.5 mg/kg). The results showed that FK treatment had significantly attenuated the inflammation induced by DNCB. The increased concentration of cytokines including IL-6, IL-18, and IFN-*γ* in ACD rats could be reversed by the FK administration, while IL-10 expressed the opposite result; the expression level of TLR4, pERK_1/2_, and NF-*κ*B could be downregulated by the treatment with FK in the ACD rat. In a word, the total flavonoids of the FK had an anti-inflammatory effect on the DNCB-induced ACD rat; this regulatory mechanism was highly possible based on the pERK_1/2_/TLR4-NF-*κ*B pathway activation.

## 1. Background

Fructus Kochiae Scopariae (Fructus Kochiae, FK) is the desiccative ripe fruit from* Kochia scoparia* (L.) Schrad., which has been used as a traditional Chinese medicine for a long time. The traditional efficacies of FK were recorded in ancient medicine books, like* Herbal Canon*. The bioactive components of FK were progressively isolated and analyzed along with the in the blossom of herbal pharmacology. The FK extraction had shown an inhibition effect on the scratching behavior, oleanolic acid oligoglycoside and momordin Ic isolated from this extraction also exhibited the antipruritogenic effect [1]. In addition, these two components had been testified to be an effective way to alleviate the carbon tetrachloride-induced hepatotoxicity in rats [2]. There was a peripheral antinociceptive effect after the animals were orally treated with the 70% ethanol extraction of FK, this process might be mediated by the anti-inflammatory activity of momordin Ic. Moreover, the n-butanol fraction from FK had been examined that it can reduce the serum glucose level by restraining the transport and transformation of sugar in the digestive tract [3]. The chloroform fraction of FK had been proved to have an acaricidal activity [4]. The flavonoids of FK have been isolated to examine its content and antioxidant activity [5], but rarely on the anti-inflammatory effect.

Allergic contact dermatitis (ACD) is a delayed-type hypersensitivity rising from the contact of an allergen broken out between the sensitive individuals. This disease actually mediated by T lymphocyte cells, the combination of sensitized T lymphocyte, and its corresponding antigen binding leads to an activation of the release of a variety of cytokines, forming an inflammation action characterized by infiltration of monocytes and tissue degeneration. Due to its susceptibility, iterancy, and universality of paroxysm, it has been an intractable disease in the clinical treatment. The routine therapy of ACD depends on antibiotics, steroids, and chemical drugs, which may bring out obvious harmful side-effects [6–8]. Therefore, specific efficient and low-toxicity substitutes for ACD have become a buzzing field. A large amount of researches have been devoted to the curative effect of Chinese herbal medicine. Sung et al. [9] used TMA intervention to modify an ACD mouse model and discussed the pharmacological effects of Achyranthis radix extract, which was proved to be an effective agent for treatment of ACD disease, the anti-inflammatory mechanism was that Achyranthis could attenuate the II-1*β* and TNF-*α* level in the ACD mouse body. Similarly, oral administration of paeoniflorin was demonstrated to attenuate ACD by inhibiting dendritic cell migration and Th1 and Th17 differentiation in a mouse model [10]. Sun et al. [11] found that the* Eriobotrya japonica* seed extract could increase the IL-10 and reduce the IFN-*γ* in the ACD rat. Their researches have demonstrated that the mechanism of antiallergic action relied on the balance of Th1/Th2 in allergic dermatitis. The elaboration of these anti-inflammatory mechanisms on ACD has concentrated on the regulation of lymphokine from T lymphocyte (IL-2, IL-4, IL-10, IFN-*γ*, and TNF-*β*) and monokine from monocytes (IL-1, IL-6, and TNF-*α*) [12]. The maturation of dendritic cells (DCs) and the differentiation of T cells were alternatives to the way of this problem [13]. However, the pharmacological action of the flavonoids of FK remains obscure; relative research is still barely illuminated by scientific literature.

It has been widely reported that the NF-*κ*B pathway was involved in the regulating mechanism of the anti-inflammatory effect. Malvidin, a flavonoids derivative, had been suggested to display an anti-inflammatory effect on cells via the inhibition of NF-*κ*B expression [14]. Recently, a research based on the DINP-induced ACD mice model reported that the TRPA1 had a decisive role in aggravating the inflammation via the NF-*κ*B activation [15]. Generally, the inflammatory response is a multiply-chemokine network in which many inflammatory factors participated. The key cell signal conducting molecules like the transmembrane receptor ERK and TLR4 have also been widely discussed in the pathological mechanisms of ACD as they are closely relative to inflammation [16–18]. In this paper, a DNCB-induced ACD rat model was utilized to explore the anti-inflammatory effect of the total flavonoids of FK. To evaluate therapeutic efficiency, four aspects were conducted: the quality of FK, the construction of ACD model, the traits evaluation, and biochemical analysis of the inflammatory molecules. Then, the serum-concentration of IL-6, IL-10, IL-18, and IFN-*γ* was investigated to show the cytokine levels. A combination of immunohistochemistry and western blot was used to reveal the change of TLR4, pERk_1/2_, and NF-*κ*B p65 in the pathogenic skin.

## 2. Methods

### 2.1. Reagents and Materials

1-Chloro-2,4-dinitrobenzene (DNCB), acetone, olive oil, and chloral hydrate were obtained from Sinopharm Chemical Reagent Co. (Shanghai, China). Sodium carboxymethylcellulose, absolute ethanol, 4% paraformaldehyde (PFA), xylene, ethylenediaminetetraacetic acid (EDTA), and hematoxylin and eosin were purchased from Wuhan Goodbio Technology Co., Ltd. (Wuhan, China). Prednisone acetate tablets were obtained from Anhui Jintaiyang Pharma (Anhui, China). Enzyme-linked immunosorbent assay (ELISA) kits were purchased from Nanjing Jiancheng Co. (Nanjing, China). Fructus Kochiae was purchased from Anhui Xiehecheng Co., Ltd. (Hehui, China), the place of origin was Henan, and batch code was 15112601. Chromatographically pure acetonitrile was purchased from Tedia (USA). Rutin, batch no. 3920, purity 99.3%, and quercetin, no. 33467, purity 98.6%, were obtained from Shanghai Shidan Biotechnology Co., Ltd. (Shanghai, China).

### 2.2. Animals and Ethics Statement

Sprague-Dawley rats were purchased from Hubei Center for Diseases Control and Prevention, weighing 50–60 g with half female and half male. The rats were housed under a specific pathogen free (SPF) level in the laboratory animal center of South-Central University for Nationalities. Each group of animals had access to the same food and water ad libitum. All animal anti-inflammatory experiments on conscious animals were performed with the NIH guide for the care and use of laboratory animals and Ethical Issue of the IASP [19].

### 2.3. Preparation of the Total Flavonoids of Fructus Kochiae

200 g FK was added to the 80% ethanol for conventional heating reflux extraction for three times, the volume of ethanol for each time was 1000 ml, and each process lasted for 60 min. After the extractions were merged of all the three times, we should dispose mixed liquid with the vacuum concentration rotary evaporator at 60°C until it was no longer with ethanol smell. Preprocessed D101 macroporous adsorption resin 200 g was mixed with the concentrated extraction for 24 h of static adsorption, then a gradient elution in the 1.0 cm chromatographic column was carried out with the water and gradient (10%, 20%, 30%, 40%, 50%, 60%, 70%, 80%, 90%) ethanol at a flow rate of 1.0 ml/min, with 6 times column volume. The effluent was collected in a unit of one-column volume. Then we measured the total flavonoids concentration with UV at 510 nm and calculated the yields. The rutin and quercetin in the total flavonoids of FK were under the quantitative analysis with the method of HPLC-DAD. The chromatographic condition was as follows: Agilent TC-C18 (4.6 mm × 250 mm, 5 *μ*m), mobile phase A was acetonitrile and B was 0.2% phosphoric solution. DAD detection wavelength was 200–400 nm, velocity of flow was 1.0 mL/min, sample size was 10 *μ*L, and column temperature was 25°C. Elution program was 0~20 min, A18%; 20~35 min, A18% → 50%; 35 min~40 min, A50%.

### 2.4. Rat ACD Model Construction and Treatment

**Figure 1:**
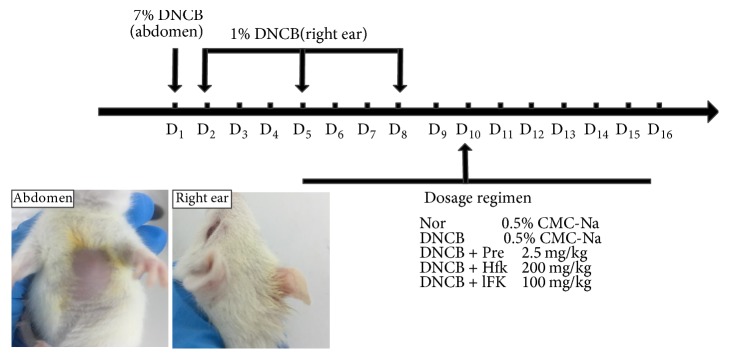
A brief diagram of the ACD model construction and treatment protocol.

We used DNCB to induce a rat ACD model according to a diffusely employed protocol [20, 21]. The DNCB was dissolved in a mixed solvent (acetone/olive oil, 4 : 1, v/v); 7% and 1% DNCB solution were prepared, respectively. After the abdominal skin was shaved with a clipper, we used the sodium sulfide to completely remove the hair in the area of 2 cm^2^. Firstly, 50 *μ*l of 7% DNCB solution was applied to sensitize the rat on the first day, and it was continually given 50 *μ*l of 1% DNCB solution for a reinforcing sensitization on the next day. 20 *μ*l of 1% DNCB solution was evenly coated on the right ear of rat on the fifth day, and a repetitive operation was carried out on the subsequent three days. The drug administration started on the fifth day with a frequency of twice a day; all the drugs were dissolved in the 0.5% CMC-Na. There are five groups in our experiments: the DNCB group (DNCB) and the normal group (Nor) were treated with 0.5% CMC-Na; DNCB + hFK group was treated with a high dose of total flavonoids of FK (200 mg/kg) while the DNCB + lFK group was treated with a low dose of FK (100 mg/kg); the DNCB + Pre group was treated with prednisolone acetate (2.5 mg/kg) and it was taken as the positive control group [22]. A brief diagram of the ACD model construction and treatment protocol was shown in Figure 1.

### 2.5. Histological Evaluation

After ten days of drug therapy, the histological allergic action of the right ear was observed according to the Eczema Area and Severity Index (EASI) scoring system [23]. The ear thickness was measured by a digimatic micrometer. A punched piece of the right ear (diameter 8 mm) was taken out by a perforator; the ear swelling was measured by the electronic scale. The ear tissues were fixed with 4% PFA and, meanwhile, embedded them in paraffin and cut into slices (4 *μ*m) for hematoxylin-eosin (H&E) staining. Then, sections were examined and photographed, and the original magnification was 100x and 200x, respectively. The monocyte infiltration and epidermal keratinization were taken into evaluation.

### 2.6. Enzyme-Linked Immunosorbent Assay (ELISA)

After ten days of drug administration, the blood samples of the rat were collected; before the centrifugation (3000 rpm for 10 min, 4°C) we had to wait for 30 min at the room temperature. Then we collected the serum samples and stored them at −80°C. The concentrations of IL-6, IL-10, IL-18, and IFN-*γ* were quantified by ELISA analysis according to the normal laboratory protocol in the ELISA kits. The sensitivity of these assays is 1.0 pg/ml. The 6 mm diameter ear tissues were washed out with 0°C 1x PBS and then fully grinded for 5 min in ice bath with the glass homogenizer and 5 ml 1x PBS, all the samples were centrifuged (3000 rpm for 10 min, 4°C), and supernatants were obtained for ELISA analysis.

### 2.7. Immunohistochemical Evaluation

We used a classical paraffin section immunohistochemical method to evaluate the expression level of NF-*κ*B (p65), pERK_1/2_, and TLR4 [24]. The primary antibodies, which were specific for NF-*κ*B (p65), pERK_1/2_, and TLR4, were obtained from Wuhan Goodbio Technology Co., Ltd. (Wuhan, China). So was the DAB reagent kit. The second antibody was purchased from DAKO Co. (CA, USA). The integrated optical density (IOD) values of each section sample were obtained from its five fields by Image-Pro Plus 6.0, the original magnification of which was settled as 200x. The data was presented as the ratio of the IOD value of each sample to the average IOD value of the norm group.

### 2.8. Western Blot

The 6 mm diameter right ear tissue was took out by utilizing sterilized puncher and was immediately stored at −80°C. These tissue samples were cleaned with PBS before being homogenized with lysis buffer. Oscillate and ice bath were completely cracking for 30 min. The homogenate was centrifuged at 12,000 ×g for 5 min at 4°C. Then, the supernatant samples were collected, and the protein concentrations were determined by BCA protein assay kit. The proteins were separated on 12% SDS-polyacrylamide gel (SDS-PAGE) and then transferred onto the polyvinylidene difluoride (PVDF) membrane. The immunoreaction process consisted of 1 h blocking the nonspecific site with 5% nonfat dry milk and 12 h incubating with specific primary antibody at 4°C. Subsequently, the membranes were washed with tris-buffered saline with Tween (TBST) and followed by incubation with the secondary antibody at room temperature for 30 min. We used isometric ECLA and ECLB to develop and fix. All the reagents and materials that were not mentioned in Section 2.1 were obtained from Wuhan Goodbio Technology Co., Ltd. (Wuhan, China). The data was disposed with Photoshop and Image-J.

### 2.9. Statistical Analysis

All data are expressed as means ± standard deviation (SD). One-way analysis of variance (ANOVA) along with Dunnett's *t*-test was performed for statistical evaluation. Statistical analysis was conducted by using SPSS 16.0. Difference was considered as statistically significant when *p* < 0.05. For each group, the number of animals was *n* = 8~10.

## 3. Results

### 3.1. HPLC Fingerprint of the Rutin and Quercetin in the Total Flavonoids of FK

**Figure 2:**
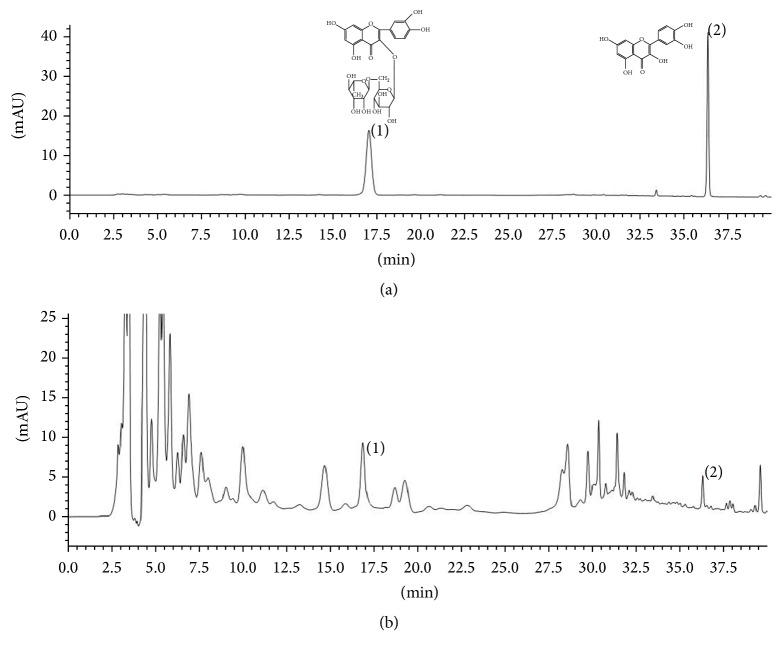
The HPLC chromatogram of the standard substance (a) and the total flavonoids of FK (b).

The results of the preparation of the total flavonoids of FK demonstrated that the flavonoids were mainly concentrated in the effluent fraction of 30%, 40%, and 50% ethanol. So we mixed the effluents of these three fractions and calculated the yield after drying under reduced pressure at 60°C. There was 2.35 g dry extract. The total flavonoids in this dry extract were detected by using rutin and quercetin as comparison samples. The yield of the total flavonoids of FK was 85.64%, which was measured with aluminum nitrate as chromogenic agent. The HPLC chromatogram of the standard substance (rutin and quercetin) and the total flavonoids of FK were, respectively, shown in Figure 2, *λ* = 370 nm. The content of rutin in the total flavonoids of FK was 24 mg·g^−1^; the content of quercetin in the total flavonoids of FK was 18 mg·g^−1^.

### 3.2. Effect of the Total Flavonoids of FK on the DNCB-Induced ACD Ear Swelling

**Figure 3:**
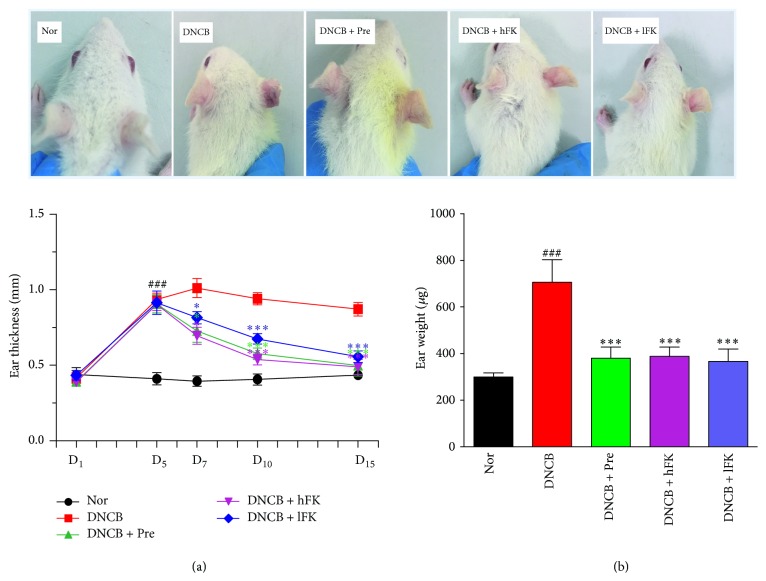
Effect of the total flavonoids of FK on the DNCB-induced ACD ear swelling. The groups treated with FK showed significant swelling recover at day 15. (a) The ear thickness of rat at days 1/5/7/15: the groups treated with FK showed significant ear thickness attenuation since day 7. (b) The ear weight of rat at day 15: the groups treated with FK showed ear swelling recovery at day 15. The data were present as the means SD (*n* = 10). Compared with the Nor. group, ^###^*p* < 0.001; compared with the DNCB group, ^*∗∗∗*^*p* < 0.001,^*∗*^*p* < 0.05.

After ten days of treatment, the five groups of rats showed different symptoms. Compared to the norm group, the right ears of rat in DNCB group displayed varying degrees of grievous swelling, redness, and scaling. Compared to the DNCB group, the tissue lesions of each treatment group conferred a remarkable turnaround. The efficacy of group DNCB + Hfk and DNCB + lFK had no difference with the DNCB + Pre group. The ear thickness and ear weight were analyzed which served as another ways to count the ear swelling. The results demonstrated that the ACD model construction method was quite stable and feasible. After ten days of drug treatments, the ear thickness and weight had a significant improvement for high dose and low dose FK administration, and the therapy of FK was no better or worse than prednisolone acetate administration. The detailed results were shown in Figure 3.

### 3.3. Effect of the Total Flavonoids of FK on the DNCB-Induced ACD Histologic Changes

**Figure 4:**
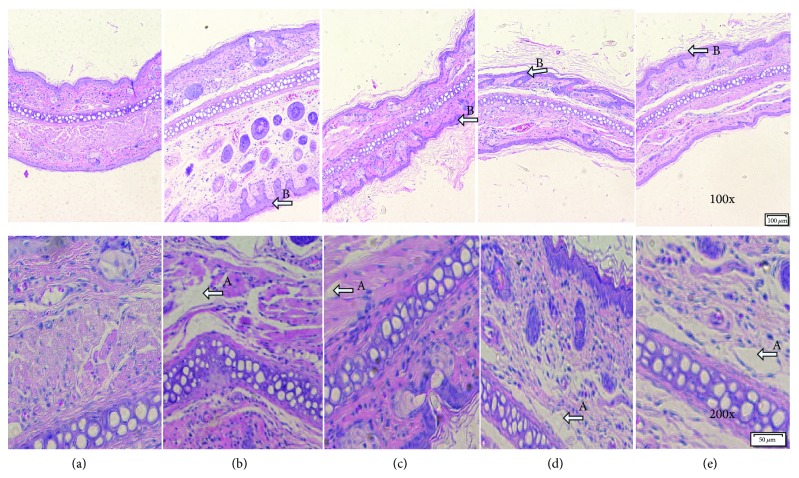
Effect of the total flavonoids of FK on ear histopathology on the DNCB-induced ACD rat (original magnification 100x and 200x). (a) Nor., (b) DNCB, (c) DNCB + Pre, (d) DNCB + hFK, and (e) DNCB + lFK. The marked sites with arrow: A denoted the monocyte infiltration and B denoted the epidermal keratinization.

Compared to the norm group, DNCB induced an ACD rat model that was characterized by a significant monocyte infiltration and epidermal keratinization. Visually, the HE slice of DNCB group showed a severe swelling, and, after ten days of drug administration, FK and prednisolone acetate inhibited this swelling observably. The amplificatory picture showed change of monocyte infiltration and epidermal keratinization in the tissue, the rats administered FK and prednisolone acetate presented a markedly inhibition of the increasing of monocyte numbers and epidermal keratinization in the right ear. The hematoxylin-eosin (H&E) staining results were shown in Figure 4.

### 3.4. The Total Flavonoids of FK Manipulated the IL-6, IL-10, IL-18, and IFN-*γ* Levels, Respectively, in Serum and Ear Tissue

**Figure 5:**
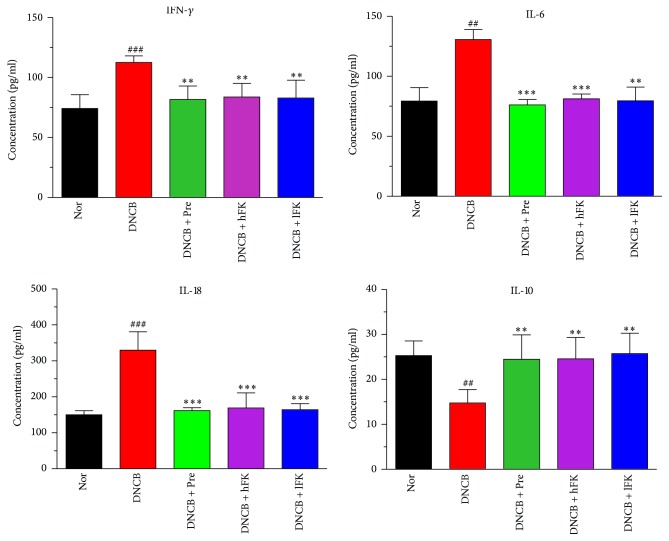
Influences of total flavonoids of FK on the IL-6, IL-10, IL-18, and IFN-*γ* levels in the serum of ACD rat. The data was presented as the mean ± SD (*n* = 6). Compared with the Nor. group, ^###^*p* < 0.001, ^##^*p* < 0.01; compared with the DNCB group, ^*∗∗∗*^*p* < 0.001, ^*∗∗*^*p* < 0.01.

The ELISA results demonstrated that the DNCB intervention could upregulate the cytokines IL-6, IL-18, and IFN-*γ* in the serum. This upregulation effect was reversed after ten days of FK administration, and the efficacy was roughly the same with the prednisolone acetate administration. Meanwhile, the concentration of IL-10 had a downregulation expression on the ACD rat, which also could be reversed after ten days of FK administration as well as prednisolone acetate administration. The remarkable statistical data was shown in Figure 5.

**Figure 6:**
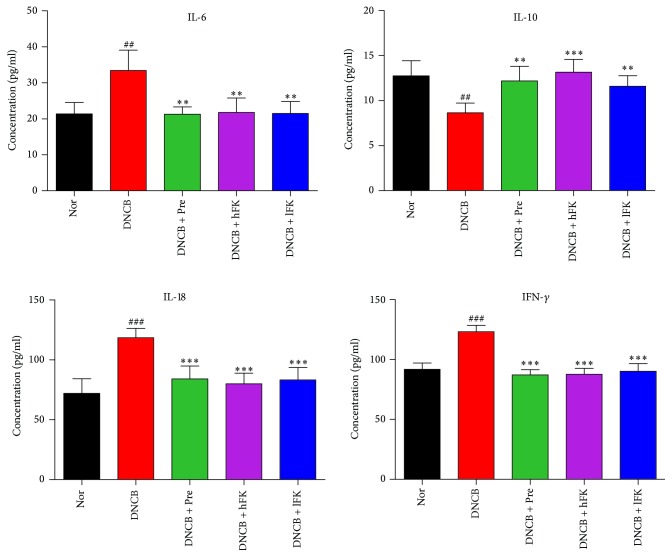
Influences of total flavonoids of FK on the IL-6, IL-10, IL-18, and IFN-*γ* levels in the ear tissue of ACD rat. The data was presented as the mean ± SD (*n* = 8). Compared with the Nor. group, ^###^*p* < 0.001, ^##^*p* < 0.01; compared with the DNCB group, ^*∗∗∗*^*p* < 0.001, ^*∗∗*^*p* < 0.01.

The ELISA results of the ear tissue showed the same efficacy trend that the IL-6, IL-10, IL-18, and IFN-*γ* levels in the ear tissue could be manipulated by the total flavonoids of FK. The detailed data was shown in Figure 6.

### 3.5. Effect of the Total Flavonoids of FK on in TLR4/pERK_1/2_-NF-*κ*B Activation in ACD

**Figure 7:**
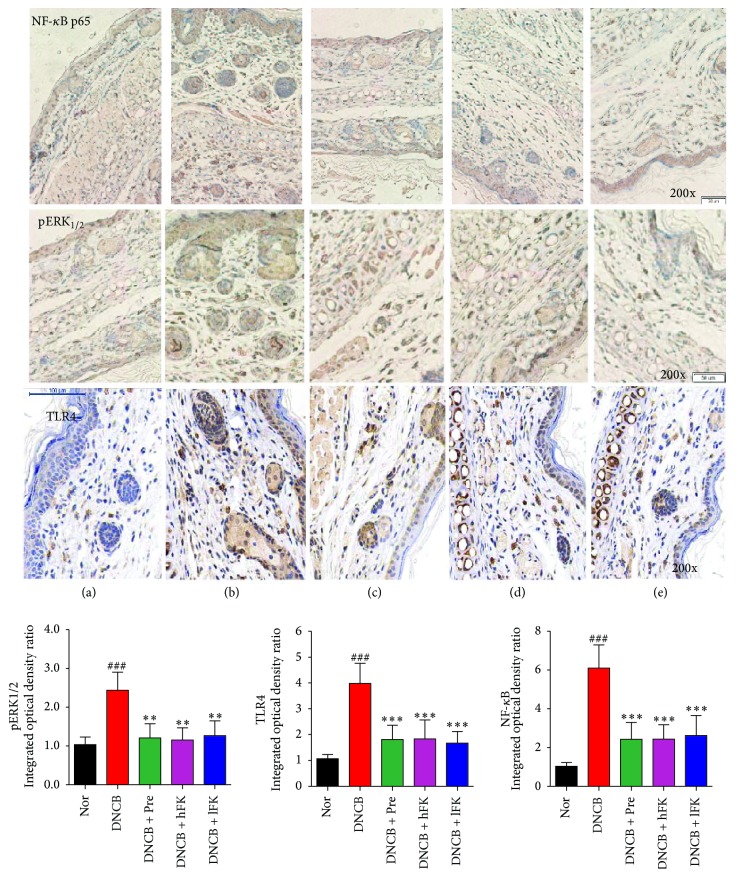
Effects of the total flavonoids of FK on in pERK1/2, NF-*κ*B, and TLR4 activation in ACD rat. The representative immunohistochemical experiments were shown; the data was presented as the mean ± SD (*n* = 5). Compared with the Nor. group, ^###^*p* < 0.001; compared with the DNCB group, ^*∗∗∗*^*p* < 0.001, ^*∗∗*^*p* < 0.01.

**Figure 8:**
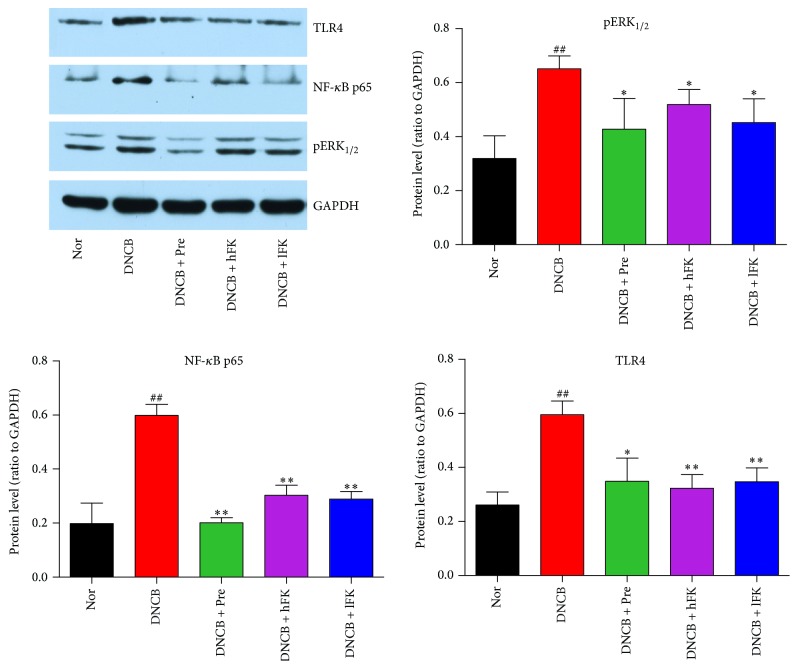
Effects of the total flavonoids of FK on in pERK_1/2_, NF-*κ*B, and TLR4 activation in ACD rat. The representative western bolts were shown; the data was presented as the mean ± SD (*n* = 3). Compared with the Nor. group, ^##^*p* < 0.01; compared with the DNCB group, ^*∗∗*^*p* < 0.01, ^*∗*^*p* < 0.05.

The immunohistochemical experiment result was shown in Figure 7. The western bolt experiment displayed the same tendency as immunohistochemical experiment, and this result was shown in Figure 8. These results illustrated a potential mechanism of effect of the total flavonoids of FK on ACD rat, an anti-inflammatory activity based on the activation of the pERK_1/2_-TLR4-NF-*κ*B signal pathway. The protein expression level of TLR4 (Toll-like receptor 4 was a single membrane-spanning and noncatalytic receptor that is usually expressed on sentinel cells) increased along with the DNCB intervention on the right ear, and this augmentation could be downregulated by the treatment of FK. The results of pERK_1/2_ (phosphorylated extracellular regulated protein kinases 1/2, the key to transmit signals from surface receptors to the nucleus) analysis showed the same expression profile, as it was upregulated by the activation of TLR4. Then the signal modulation came to NF-*κ*B (nuclear factor-*κ*B, a nuclear transcription factor); the inflammation caused by DNCB smear finally resulted in an increased NF-*κ*B p65 expression. Compared to the norm group, the expression quantity of NF-*κ*B p65 in DNCB group showed a significant increase. Compared to DNCB group, the FK treatment leaded to a remarkable inversion effect of this upregulation.

## 4. Discussion

The morbidity of ACD always owns to the contact of the anaphylactogen, which can stimulate the skin and force the body to immune response. The chief anaphylactogen of the ACD comes to some low molecule weight chemicals such as such as thiuram, nickel (Ni^2+^), epoxy and other resins, aromatic amines, and chromate [25]. In this study, we used DNCB as the allergen exposure to induce the ACD model. DNCB is a hapten that can integrate the soluble fraction of epithelial protein to be the complete antigen. The acetone solution of DNCB has been widely used to smear on the skin of rat or mouse in the ACD researches [26–28]. The pathogenic mechanism of DNCB-induced ACD was reported to be T cells infiltration in the skin, which is characteristic of Th1-induced delayed-type hypersensitivity response [29] and Th2-induced allergic response [30]. Actually, the differentiation of Th1 cells and Th2 cells depends on the cytokines they are exposed to; in the DNCB-induced ACD animal model, cytokine IL-18 and IL-10 induce Th1 differentiation while IL-4 causes Th2 differentiation and antagonizes Th1 development [31, 32]. Besides, it is well known that the cytokines related to ACD also include IL-1, IL-2, IL-6, and IL-17 and IFN-*α* and IFN-*γ* [12, 14, 33]. It is plain to see that ACD is a complicated process with numerous regulation factors to participate in.

To find out the pharmacological mechanism of the anti-inflammatory effect of FK, we used the cytokines IL-6, IL-10, IL-18, and IFN-*γ* in the ear tissue and serum to evaluate the mediation function of FK on the balance in the Th1/Th2 ratio. The present results had verified that the DNCB intervention could upregulate the IL-6, IL-18, and IFN-*γ* induced Th2 differentiation while the downregulation of IL-10 showed a suppression effect on the Th1 activation. Treatment with the total flavonoids of FK, which had been confirmed as containing rutin and quercetin, significantly inhibited these imbalanced secretion of IL-6, IL-18, and IFN-*γ* and increased the IL-10 production by lymphocytes, which strongly suggested that the total flavonoids of FK had a therapeutic effect on ACD via ameliorating the inflammatory cytokine secretion of Th1/Th2 cells.

Another nonnegligible inflammatory signal transduction factor is the signal protein. The extracellular signal-regulated kinase (ERK) contains two similar (85% sequence identity) protein kinases which were originally called ERK1 and ERK2 [34]. They are rapidly phosphorylated after the cell surface tyrosine kinases such as the epidermal growth factor receptor activation and this reaction is quite common in the inflammation disease [35]. Members of the toll-like receptor (TLR) gene family also convey signals stimulated by these inflammatory factors, activating signal transduction pathways that result in transcriptional regulation and stimulate immune function [36]. Nuclear factor-*κ*B (NF-*κ*B) is a nuclear transcription factor that regulates expression of a large number of genes that are critical for the regulation of apoptosis, inflammation, and various autoimmune diseases. The activation of NF-*κ*B is thought to be part of a stress response as it is activated by a variety of stimuli that include growth factors, cytokines, lymphokines, and pharmacological agents [37]. Relative researches had demonstrated the NF-*κ*B activation response to the cytokines [38], ERK activation [35], and TLR4 activation [39], while most of study of NF-*κ*B system concentrated on the discussion of IL-1 and TNF-*α*; those had been widely confirmed as the key initiating factor for NF-*κ*B pathway in the inflammation response of ACD [40–42].

To investigate the expression of NF-*κ*B and the role of which in the ACD rat, the present study focused on the elaboration of inflammation signal transmission via related signal protein molecule pERK_1/2_, TLR4, and NF-*κ*B p65. Combined with analysis of the results of immunohistochemistry and western blot, the protein expression level suggested that the ERK was rapidly phosphorylated after the cell surface contact with the hapten DNCB. This process might also be reinforced because of the secretion of cytokines which had been mentioned above. The increasing of TLR4 expression also accelerated the signal transduction to the NF-*κ*B system and finally induced a graver inflammatory response. The total flavonoids of FK showed a suppression of NF-*κ*B p65 expression via attenuating the ERK/TLR4 signals.

However, in comparison with the total flavonoids of FK, the prednisolone acetate administration showed a slight superiority. And the FK extract still contacts too many physiological active substances, some of which, like rutin, had been proved to have anti-inflammatory effect on the ACD mice [43]. A more straightforward structure-function relationship and pharmacological mechanism might still be a meaningful research. Our current work had established this foundation that the total flavonoids of FK had significant anti-inflammatory in the ACD rat.

## 5. Conclusions

In this study, oral administration of the total flavonoids of FK displayed a significant anti-inflammatory activity in ACD rat, including the inhibition of monocyte infiltration and epidermal keratinization and mediation of cytokines in ear tissue and serum. The potential mechanism of this effect owned to the suppressing of NF-*κ*B expression via the ERK/TLR4 signal pathway.

## Abbreviations

ACD: Allergic contact dermatitis
FK: Fructus Kochiae
DNCB: Dinitrochlorobenzene
EASI: Eczema Area and Severity Index
ELISA: Enzyme-linked immunosorbent assay
H & E: Hematoxylin and eosin
IL: Interleukin
IOD: Integrated optical density
PFA: Paraformaldehyde
EDTA: Ethylenediaminetetraacetic acid
PBS: Phosphate-buffered saline
SD: Standard deviation
NF-*κ*B: Nuclear factor-kappa B
TLR: Toll-like receptor
ERK: Extracellular signal-regulated kinase.
